# Sumatriptan and dihydroergotamine in proximal and distal human isolated coronary arteries

**DOI:** 10.1186/1129-2377-1-S14-P206

**Published:** 2013-02-21

**Authors:** S Labruijere, MB Ramirez Rosas, R De Vries, AHJ Danser, A MaassenVanDenBrink

**Affiliations:** 1 Erasmus Medical Center, Netherlands

## 

Sumatriptan and dihydroergotamine (DHE) are both 5-HT receptor agonists and two of the most widely used drugs for the acute treatment of migraine. These drugs are contra-indicated in people with cardiovascular disease because of their vasoconstricting properties, as has previously been assessed in proximal coronary arteries. The effect of DHE in distal coronary arteries, however, has never been reported, although smaller coronary arteries might also account for angina-like symptoms, especially in women. The aim of this study was to compare the contractile effects of sumatriptan and DHE in proximal and distal human coronary arteries, and to relate our findings to the plasma concentrations obtained in clinical practice. Segments of proximal (Ø 3-5 mm) and distal (Ø 0.5–1 mm) human isolated coronary arteries were mounted in organ baths and concentration response curves for sumatriptan and DHE were constructed. In proximal coronary artery segments, maximal contractions to sumatriptan (16+/-18% of contraction to 100 mM KCl) and DHE (5+/-4%) were not significantly different. In contrast, in distal coronary arteries, the contractile responses to sumatriptan (18+/-11%) were significantly larger than those to DHE (4+/-2%). At clinically relevant concentrations (Cmax after different formulations), contractions to both sumatriptan and DHE in proximal as well as distal coronary arteries were below 6%. Thus, our results indicate that coronary artery contractions to DHE in distal coronary artery are smaller than those to sumatriptan, although in the clinical situation both drugs are likely to induce only a slight contraction.
